# Links between self-injury and suicidality in autism

**DOI:** 10.1186/s13229-020-0319-8

**Published:** 2020-02-10

**Authors:** R. L. Moseley, N. J. Gregory, P. Smith, C. Allison, S. Baron-Cohen

**Affiliations:** 1 Department of Psychology, Bournemouth University, Poole, UK; 2 Autism Research Centre, Department of Psychiatry, University of Cambridge, Cambridge, UK

**Keywords:** Autism, Self-injury, Suicidality

## Abstract

**Background:**

Autistic individuals without intellectual disability are at heightened risk of self-injury, and appear to engage in it for similar reasons as non-autistic people. A wide divergence of autistic perspectives on self-injury, including those who frame it as a helpful coping mechanism, motivate investigating the link between self-injury, suicide ideation, and attempts which has been reported in typically developing individuals.

**Method:**

One hundred three autistic participants completed the Non-Suicidal Self-Injury Assessment Tool (NSSI-AT), the Suicide Behaviors Questionnaire (SBQ-R), and the Interpersonal Social Evaluation List (ISEL-12) across two online studies. Logistic regression was conducted to predict self-harming status via responses to questions on suicidality, and to predict whether certain self-injurious behaviors, including cutting, were especially associated with suicide ideation and attempts. Non-parametric correlation analysis examined relationships between suicide ideation/attempts and other variables that might characterize self-harmers especially at risk of suicidality. These included perceived access to social support, purposes or reasons for self-injury, the number of different self-injurious behaviors engaged in, the duration and lifetime incidence of self-injury, and the individual’s feelings about their self-injury.

**Results:**

While self-injuring status was significantly predicted by responses to a question on suicide ideation and attempts, there was no relationship between suicide ideation/attempts and a participant’s personal feelings about their self-injury. The method of cutting was also predicted by suicide ideation and attempts, though other methods common in autistic people were at borderline significance. Use of self-injury for the regulation of low-energy emotional states like depression, for self-punishment or deterrence from suicide, and for sensory stimulation, was associated with suicide ideation and attempts, as was the number of self-injurious behaviors engaged in. There was no significant relationship between suicide ideation/attempts and the duration and lifetime incidence of self-injury or social support.

**Conclusions:**

These preliminary data suggest that while individuals might frame their self-injury as a positive or neutral thing, there remains a concerning relationship between self-injury and suicidality which exists regardless of individual feelings on self-injury. This is consistent with the theoretical perspective that self-injury can be a “gateway” through which individuals acquire capability for lethal suicidal behaviors. The data highlight that particular methods (cutting) and reasons for self-injury may be of significant concern, but this information, which might be of extreme value for clinicians, requires further investigation and validation.

Non-suicidal self-injury (NSSI; otherwise known as self-mutilation or self-harm) describes the causing of deliberate physical injury to the body without suicidal intent [1]. Self-injurious behaviors are diverse, with such examples as cutting, scratching, burning, or hitting oneself. From the outside, these behaviors are counterintuitive, bizarre, and frightening to the onlooker, but self-injury is believed to fulfill particular functions, or meet certain needs, which an individual may only partially understand [1]. From this approach, self-injury is a negatively or positively reinforced behaviour which functions^1^ “as an immediately effective method of regulating one’s affective/cognitive experience and/or influencing one’s social environment” (Nock 2010, p.10). Accordingly, NSSI may serve at an interpersonal level to influence the behavior of others (for instance, to *stop* a parent’s demands) or to communicate or express one’s own distress (e.g., to *gain* affection). Intrapersonally, it may be positively or negatively reinforced as a private means of sensory stimulation or emotion regulation, for instance through the satisfaction an individual gains from self-punishment. NSSI may have different functional roles (i.e., be performed for different reasons) depending on the occasion [1–5], and may sit alongside other maladaptive behaviors (such as substance abuse and disordered eating) which function in the same way and meet the same needs. Conversely, while some individuals report engaging in self-injury as a means of avoiding more dangerous, suicidal behaviors, NSSI is closely related to suicidal thoughts and behavior [6–15]. It appears to be a “time-invariant” or longitudinal predictor of suicide attempts [12], with authors suggesting that the capacity for self-injury is a “gateway” to suicidal behaviors [16], perhaps through increased tolerance to pain, reduced fear of death, and increased desire for death, habituation to the effects of self-injury, or other mechanisms [17–19]. Indeed, the current leading model of suicidality [20, 21] suggests that acquired capability for suicide is required to transform suicidal intent into suicide readiness and attempts. Though provocative and painful incidents (such as violent incidents and abuse) are one means of acquiring capability for suicide through increasing tolerance for pain and fearlessness of death, NSSI is one of the most direct mechanisms by which people move from suicidal intent to being capable of lethal attempts [22, 23].

One population at especial risk of suicidality is people with autism spectrum conditions (ASC; hereafter “autism”) [24–32]. ASC are neurodevelopmental conditions characterized by difficulties with social communication and relationships, and by repetitive and restricted patterns of behaviour and interests [33]. Tragically, by the age of ten, autistic children are already at significantly greater risk of suicide ideation and attempts [25], and from this age the risk only increases. In the USA, the prevalence of autism was recently estimated at 1 in 68 children [34], illustrating a great need for support and intervention to address this elevated risk. Given the established link between NSSI and suicide in the general public, the fact that autistic people appear to have higher rates of suicide *and* of self-injury [35–37] may not be coincidental.

Investigations of NSSI in autism are still very limited; the majority of these pertain to the high-frequency behaviors such as head-banging and biting that are often seen in minimally verbal individuals with intellectual disabilities, and which tend to be classified as an aspect of the stereotyped repetitive and restricted behaviors inherent to the diagnosis [36–38]. These behaviors have been differentiated from the type of self-injury (NSSI) described above in the typically developing population. Only recently has self-injury been examined in autistic people without intellectual disability, the subset of the spectrum at greater risk of suicidality [29, 30, 32, 39] and whom appear to engage in self-injury in very similar ways to non-autistic people in so far as age of onset, methods used, and the functional purpose that self-injury serves [35]. The only apparent differences in this analysis were that autistic individuals were more likely to use self-injury as a means of shocking or hurting others, imitating peers, or avoiding more severe suicidal behaviors.

Our own group conducted a more in-depth analysis of self-injury in 103 autistic adults without intellectual impairment [40]. As in the previously described study [35], we corroborated an average onset age in adolescence; the most common methods of self-injury (scratching/pinching and cutting) and most common bodily locations (arms and hands); and most common initial motivations (anger at the self and upset, though a large proportion of our participants claimed to have stumbled on self-injury “accidentally” and found it served a functional purpose, i.e., in fulfilling some need). We found that the most common functional purpose of self-injury was to regulate low-energy affective states such as depression or numbness; the second most common was to regulate high-energy affective states such as anger or agitation. This was followed by use of self-injury for self-punishment and/or deterrence from suicide, for sensory stimulation, and lastly, for social communication and expression. Our qualitative analysis greatly expanded on the needs that NSSI fulfills in autistic people. Participants spoke of low confidence, low self-esteem, and indeed self-hatred in relation to self-injury. They also spoke of emotions such as anger, frustration, anxiety, and stress (or unnamed “emotional pain,” “pressure,” or “hurting inside”) as triggers for self-injury, and that being able to identify and verbalize their emotions was helpful in controlling self-injury. This interestingly corroborated our quantitative analysis, where alexithymia (an often comorbid difficulty identifying one’s own emotions) was a predictor of self-injury. Sensory issues were also identified as triggers, and were again corroborated as a predictor of self-injury in our quantitative analysis. That both alexithymia and sensory disturbances are predictive of self-injury in autistic people is consistent with the prevalence of these features in other self-harming populations, *and* with the relationship that both alexithymia and sensory differences show with internalizing symptoms and/or mental ill-health in autism [41–46]. Alexithymia is higher than average in adolescent self-harmers and is a well-known correlate of self-injury in clinical populations [47–49]. Sensory disturbances have been a topic of interest as pertains to self-injury in autistic children and teenagers with and without intellectual disability. In these groups [36, 50], much as in our adult sample, atypical sensory experiences have been seen to predict self-injurious behavior. Self-injurious behavior was also seen to be associated with alterations to somatosensory cortices [51], though these differences might reflect brain plasticity in response to repetitive self-injury rather than pre-existing and potentially causal differences. While NSSI often serves multiple functions simultaneously, self-injury to obtain sensory stimulation is often referred to as “automatically reinforcing” because regardless of changes the behavior might produce in the environment, the sensory experience it produces is reinforcing by itself [52]—for instance, by regulating hypo- or hyper-arousal [50].

Our previous study drew short of studying the link between self-injury and suicidality in autism. This relationship is currently underexplored, though there is some early evidence from Cassidy and colleagues that lifetime incidence of self-injury confers a heightened risk of suicidality much as it does in the non-autistic literature [53]. These authors, however, used a dichotomous categorization of participants as self-harmers or non-self-harmers, without consideration of whether NSSI was historic or current. Moreover, they did not consider additional nuances, such as whether the functional purposes which motivate self-injury affect the relationship between NSSI and suicidality. Following on from our previous work, we query whether the conscious perspectives or meanings *ascribed* to NSSI are also important determinants of the suicide risk faced by individuals who self-harm. Our qualitative findings [40] suggested a striking dichotomy between participants who expressed distress and/or a lack of conscious control over NSSI, and those who framed it as a conscious choice, a neutral or even positive option to deal with “overwhelming feelings,” “stressful situations,” or even “to achieve homeostasis”. This diversity was also manifest in the quantitative data, where 17% of self-harming participants did not perceive their self-injury as problematic in their lives, 14% were neutral about it, 24% saw perceived it as quite problematic, and 9% found it strongly problematic. Furthermore, asked what they wanted others to know about helping a loved one with self-injury, our participants highlighted the need to recognize individuality within the self-harming autistic population; to avoid common assumptions about self-injury; and to recognize where self-injury might be serving a functional, even positive, goal, and where automatically assuming it to be a bad thing might even be unhelpful.

As it stands, the association between NSSI and suicide attempts, robust in the neurotypical literature and emerging in the field of autism research [53], justifies concern and action on the part of clinicians, educators, and loved ones. The perplexing diversity of perspectives and reasons for self-injury in our sample, however, imply that to identify those individuals most at risk, a more nuanced examination of autistic self-harmers may be beneficial. In autistic people, does engaging in self-injury result in increased risk of suicidality *regardless* of the functional purpose that NSSI serves, and/or regardless of the meaning an individual attributes to their self-injury? Alternatively, is the risk of suicidality associated with the functional role that NSSI plays, and/or how people feel about it? There is some precedent, from non-autistic samples, that the functional purpose or reason why people engage in NSSI may indeed confer additional suicide risk. Those who engage in NSSI for emotion regulation or self-punishment have been found to be particularly at risk of suicide ideation and attempts [54, 55], as are those who engage in NSSI exclusively whilst alone—the lack of an audience for social communication or influence was interpreted by the authors as reflecting use of NSSI for emotion regulation or self-punishment [56].

There may be other variables that distinguish some self-harming individuals as being particularly at risk for suicidality. Individuals who self-injure seem to be at particular risk of suicide attempts the longer their history of NSSI, the frequency of self-injury, the greater the number of methods they use, and the less physical pain they experience [9, 14]. A weaker predictor is the method of cutting in particular [14]: cutting is believed to reflect greater exposure to physical pain and damage and thus increased capacity for suicide attempts, and is suggested to be more closely related to mental illness than other forms of self-injury such as head-banging [57]. Depression and hopelessness seem to present an additional risk, as does low self-esteem [58]; in contrast, psychological factors such as attribution style, self-forgiveness, and feelings of agency may confer resilience [59–61]. Social support, or indeed *perceived* social support, also decreases the risk of self-injury turning to suicide attempts [62]. Parental support especially reduces the likelihood of suicide attempts in young self-harmers [16, 58], and appears to be further helpful in moderating the likelihood that bullied individuals will self-injure [63]. Of course, given the prevalence of NSSI in adolescents, these investigations of that sample may not hold true for an autistic adult group, many of whom may not have parental support.

The present study attempted to extend the previous in-depth investigation of self-injury by exploring its relationship with suicide ideation and attempts. In particular, we focused on whether the presence of self-injury predicted suicide ideation or attempts; whether there was an association between how individuals felt about self-harming and suicide ideation and attempts; whether endorsement of statements about different functions that self-injury plays (or needs it fulfills) were associated with suicide ideation and attempts. We also looked at relationships between suicidality and duration of NSSI, whether range of self-injurious behaviors predict suicidality as they do in non-autistic populations, and whether participants who cut were at particular risk. As our participants were adults and unlikely to have the same level of parental support as the studies reflecting this as a moderator in teenagers, we looked instead at whether perceived social support was associated with lower suicide ideation and attempts.

## Methods

### Participants

This study was advertised to participants as an investigation of stress and physical and mental health. We invited back autistic participants from the previous investigation into self-injury [40], who had been recruited from support groups local to the primary researcher (Dorset), from social media, and from the Cambridge Autism Research Database (CARD) at the Autism Research Centre, Cambridge, UK. There was a high rate of return with 82 participants responding (80% of the original sample). We additionally advertised the study on Facebook support groups run by and for autistic people. Of those who responded to this call, 20 were willing to complete the Non-Suicidal Self-Injury Assessment Tool (NSSI-AT) as an optional extra, such that we possessed data from the three measures needed in the current analysis.

In total, therefore, 102 autistic participants were included in the analysis. The group consisted of 29 males and 73 females, with an overall average age of 42.6 years (SD 14, range 54). The average age at diagnosis was 34 years (though there was a large SD of 17.2 years). The majority of participants, 66, were British (65%), after which the biggest minority was American (11 participants, 11%); the remaining participants hailed from Australia, New Zealand, Canada, Germany, the Netherlands, Finland, Ireland, Hungary, Venezuela, and the Czech Republic. Unfortunately, due to the nature of the study, diagnoses could not be independently verified by the research group, but participants reported the date, location, and precise diagnosis given, along with any additional diagnoses.

IQ measures were not obtained, but it is highly likely that all participants were in the range of normal to high IQ: all but two participants (2%) were qualified to at least GCSE-level, and 62 (61%) had a degree. The late average age of diagnosis further corroborates the probability that participants had cognitive skills (and, potentially, camouflage skills) at the level that allowed them to elude diagnosis as children, members of the “lost generation” described by Lai and Baron-Cohen (2015). Fifty-one (50%) of the participants were employed at the time of the study, and 12 (12%) were involved in a voluntary job. The vast majority, 79 participants (77%), reported additional psychiatric diagnoses, among which the most common were depression (61 participants, 60%) and anxiety disorders including generalized anxiety, social anxiety, obsessive-compulsive disorder, specific phobias, and post-traumatic stress disorder (54 participants, 53%): scores on the Beck Depression Inventory [64] and the Beck Anxiety Inventory [65] corroborated high average scores for depression and anxiety in the group (an average of 22.4 and 22.8, respectively, scores which indicate moderate to severe depression and moderate anxiety). Other diagnoses included psychosis, eating disorders, bipolar disorder, and personality disorders. Thirteen participants reported an additional diagnosis of ADHD/ADD (13%); dyslexia, dyspraxia, or specific learning disability were reported by 13 participants (13%). Fifty participants (49%) were taking psychotropic medication at the time of the study.

### Materials

The data being collected across the course of two online studies, participants completed a number of questionnaires and tasks which will be presented elsewhere in forthcoming publications. As such, we describe in detail only those questionnaires which pertain to the present analysis: the Non-Suicidal Self-Injury Assessment Tool (NSSI-AT), the Suicide Behaviors Questionnaire-Revised (SBQ-R), and the Interpersonal Support Evaluation List 12. These were hosted on an online platform (Qualtrics) for participants to complete in their own time, with support provided over email if needed.

#### The Non-Suicidal Self-Injury Assessment Tool

As stated previously, the majority of participants [2] (82) completed this measure as part of a previous study [40] and then completed the SBQ-R and the Interpersonal Social Evaluation List (ISEL-12) in the context of the new study. The small remainder (22) completed the SBQ-R and the ISEL-12 first in the context of the new study, and were then willing to complete the NSSI-AT as an optional extra. This comprehensive clinical assessment of self-injury documents the nature and bodily location of any self-injurious behaviors; their functional purpose, i.e., an individual’s awareness of what NSSI does for them, the need that it fulfills; the recency and frequency of self-injury, and the likelihood that it will reoccur in future; the age of onset of self-injury; the severity of injuries (based on whether these did or should have received medical attention); the social and habitual routines or context around self-injurious behaviors (if, for example, individuals always make sure they are alone); the degree to which participants are habituated to the occurrence of self-injurious behavior; and whether individuals have sought therapy, their experiences in therapy, and their experiences of telling others about their self-injury. In our previous study, we categorized participants as non-self-harmers, historic self-harmers (those who had last self-harmed more than 2 years ago and judged themselves unlikely to do it again), and current self-harmers. As the literature suggests that NSSI is a time-invariant, longitudinal risk factor for suicidality, this step was deemed unjustified for the present study, and we simply categorized participants as self-harmers or non-self-harmers based on the current or historic presence of self-injurious behaviors.

The NSSI-AT includes a number of statements endorsing functional roles or purposes of self-injury. These include statements about NSSI for the purpose of regulating low-energy emotions, regulating high-energy emotions, social communication and expression, self-punishment and deterrence from suicide, and for sensory stimulation. As in previous investigations [2, 35], we collapsed responses of “strongly” and “somewhat agree” to indicate affirmation of that functional role (and thus a score of 1), and collapsed responses of “strongly” or “somewhat disagree” to indicate denial of that role (thus a score of zero). For each participant, we scored the number of statements endorsed for each functional role or purpose of NSSI.

The NSSI-AT also yields the onset of self-injury, such that we could calculate the duration of NSSI by subtracting this from a participant’s age. Of interest was also the “range” of NSSI, which we quantified by giving a score of 1 for each type of NSSI engaged in, such that higher scores indicated that participants engaged in a greater number of diverse self-injurious behaviors than individuals who consistently used one or two methods, irrespective of frequency (for instance, a person could engage in several different self-injurious behaviors, but may engage in them less frequently than a person who only engages in *one* select self-injurious behaviour). Finally, to quantify how participants felt about self-injury, we coded responses to the statement “The fact that I intentionally hurt myself is a problem in my life”: responses of “strongly disagree” received 0, “slightly disagree” received 1, “neither agree nor disagree” received 2, “slightly agree” received 3, and “strongly agree” received 4.

#### The Suicide Behaviors Questionnaire-Revised

The Suicide Behaviors Questionnaire Revised (SBQ-R) [66] allows clinicians to assess current and lifetime suicide ideation and attempts extremely quickly and concisely. The four items assess the occurrence of lifetime suicide ideation and/or attempts; the occurrence of suicide ideation in the last year; whether the person has ever confided suicidal intentions to somebody; and the estimated likelihood that they will attempt suicide 1 day. While it affords a sum suicidality score, we focused on the single item assessing lifetime suicide ideation or attempts. To the question “Have you ever thought about or attempted to kill yourself?”, “Never” received a score of 1, and “It was just a brief passing thought” a score of 2. Scores of 3 were given to participants who endorsed one of two statements describing suicide ideation (“I have had a plan at least once to kill myself but did not try to do it” or “I have had a plan at least once to kill myself and really wanted to die”). Scores of 4 were given to those who, in choosing one of two statements (“I have attempted to kill myself, but did not want to die” or “I have attempted to kill myself and really hoped to die”), indicated that they had made suicide attempts.

#### Interpersonal Support Evaluation List 12

The Interpersonal Social Evaluation List (ISEL-12) [67] yields a total score for perceived social support, which crucially affects physical and mental health and buffers the effect of stressors [68, 69]. It is comprised of individual subscales measuring availability of guidance/advice (appraisal), feelings of acceptance (belonging), and availability of concrete material or financial aid (tangible). Here, we used simply the summary score reflecting overall perceived social support.

The NSSI-AT was completed as part of the first study, and the ISEL-12 and SBQ-R as part of the second. There was, at most 6 months, between participants completing both parts.

### Analysis

A number of the variables in this investigation were nominal, including the dichotomous presence or absence of self-injury, and whether or not an individual engaged in cutting. Ordinal variables were also involved, such as the scores yielded by the SBQ-R item assessing suicide ideation or attempts (“Never” a score or 1, “passing thoughts of suicide” a score of 2, suicide ideation a score of 3, and suicide attempts a score of 4) and scores to the item in the NSSI-AT reflecting feelings about self-injury (“The fact that I intentionally hurt myself is a problem in my life”, with responses ranging on a 0–4 Likert scale from “strongly disagree” to “strongly agree”). Others, such as number of items affirmed for each of the functional roles (purposes) of NSSI and the range of self-injurious behaviors, were continuous but with a very small range; only the duration of NSSI (that is the length of time between the first and last time a person had engaged in NSSI) and scores in the ISEL-12 had considerable range as continuous variables. As such, the varying nature of these variables necessitated several analytical approaches.

Binary logistic regression was used to determine whether the presence or absence of self-injury (dependent variable, scored 1 or 0) was predicted by responses (1–4) to the suicidality item. To assess whether the presence of cutting conferred especial risk of suicidality, the presence of cutting (1 or 0) was the dependent variable of another logistic regression. In order to partly examine the specificity of this behavior to suicidality, three more logistic regressions focused on the presence (1 or 0) of severe scratching and/or pinching, hitting the self, and hitting objects, which we previously found were the most common forms of self-injury engaged in by autistic people (Moseley et al., under review). Where significant predictive relationships were seen, we conducted chi-squared tests to examine the distribution of responses between participants in these dichotomous categories.

The relationship between responses to the suicide item and a number of variables were of interest in this study. In order to examine a hypothesized relationship between the suicidality item (responses 1–4) and whether NSSI was perceived by participants to be a problem in their life (a 0–4 Likert scale, with 0 indicating no problem at all), we performed non-parametric Spearman’s rho correlation. The same method was used to explore relationships between suicidality and the number of different types of self-injurious behaviors engaged in, and between suicidality and the number of responses to the five functional roles, or needs that NSSI fulfills, which are defined in the NSSI-AT. With wider range, we used parametric correlation analysis to examine relationships between suicidality and duration of NSSI, and between suicidality and scores in the ISEL-12, reflecting perceived social support.

## Results

### Descriptive statistics

Participants were categorized as self-harmers (*N* = 77) and non-self-harmers (*N* = 25) as described above; descriptive statistics for the two groups are displayed in Table 1. Chi-squared tests (χ^2^) were used to compare the distribution of males and females, employed and unemployed participants, participants with degrees, participants with psychiatric diagnoses, and participants taking medication between groups. Analysis of variance (ANOVA) compared group averages in age, age at diagnosis, symptoms of depression (BDI scores) and anxiety (BAI scores), and autism symptomatology as measured by the AQ.

**Table 1:** Participant demographics

	Self-harmers (*n* = 77)	Non-self-harmers (*n* = 25)	Significant differences
Sex	19 male, 58 female	10 male, 15 female	Ns.
Age	41.5 (13.8)	446 (14.3)	Ns.
Age at diagnosis	32.5 (16.6)	38.6 (18.4)	Ns.
Depression scores (BDI)	23.7 (14.4)	18.8 (11.7)	Ns.
Anxiety scores (BAI)	24.6 (11.6)	17.6 (11.4)	*F* (1, 100) = 7.208, *p* = .008
Autism spectrum quotient (AQ)	39.9 (8.2)	36.1 (5.7)	Ns.
Percentage employed	49.4%	52%	Ns.
Percentage qualified to degree level	62.3%	56%	Ns.
Percentage with psychiatric comorbidities	84.4%	60%	χ^2^ (1) = 5.775, *p* = .016
Percentage taking medication	54.5%	32%	χ^2^ (1) = 3.839, *p* = .05

Demographics for self-harming and non-self-harming autistic groups. Averages are displayed with standard deviation in brackets, alongside statistical comparisons for the purpose of group comparison

Self-harming and non-self-harming participants did not differ in age, age at diagnosis, depressive symptoms, autism symptomatology, percentage employed, or percentage qualified to degree level. Males and females were equally distributed between the two groups. The self-harming group were significantly more anxious than the non-self-harming group at the time of the study (*p* = .008), and were more likely to have psychiatric comorbidities. They tended to be more likely to be taking medication, though this likelihood was only on the border of significance.

### Logistic regression: predicting suicidality via the presence of self-injury, and specific types of self-injury

**Fig. 1:**
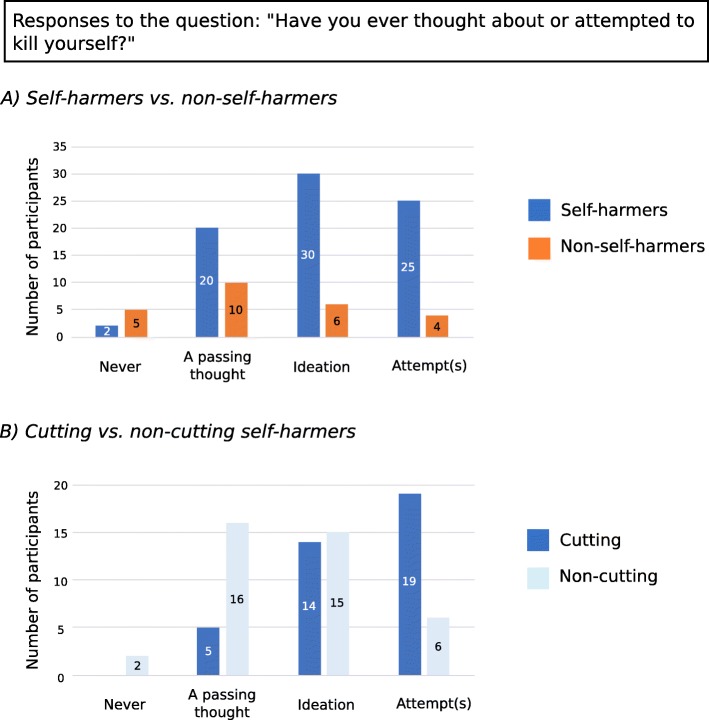
Responses of participants to the suicidality question. **a** Depicts responses of self-harming and non-self-harming participants to the question “Have you ever thought about or attempted to kill yourself?”: the number of participants who endorsed each response is shown. **b** depicts responses to the same question within the self-harming group, split into those participants who engaged in cutting and those who did not

Logistic regression revealed that responses to the question on suicide ideation or attempts were significantly different between self-harmers and non-self-harmers (χ^2^ [1] = 9.390, *p* = .002). The model, which only included responses to this question, explained 13% of the variance (Nagelkerke *R*^2^), but predicted 78% of cases correctly. The odds ratio suggested that for every 1 point increase in scores to the suicide item, the participant was 2.2 times more likely to be a self-harmer (CI 1.297, .3.848). Participants from the two groups responded as can be seen in Fig. 1a: as shown in the regression, the distribution of answers across self-harmers and non-self-harmers was significantly different (*x*^*2*^ [3] *=* 12.161, *p* = .002)*.*

In the self-harming group (*n* = 77), 38 participants reported that they cut themselves. Responses to the suicidality question significantly predicted the presence or absence of cutting (χ^2^ [1] = 15.595, *p* < .001), though the model only predicted 24% of the variance (Nagelkerke *R*^2^). Further, 67.5% of participants were correctly categorized as cutters or non-cutters based on their responses to the suicidality question, and the odds ratio suggested that for every 1 point increase in scores to the suicide item, participants were 3.3 times more likely to report cutting (CI 1.713, 6.442). The different distribution of cutters to each response of the suicide question was confirmed by a chi-squared test (*x*^2^ [3] = 14.546, *p* = .002), which showed that cutters tended to be distributed toward the more severe responses indicating suicide ideation or attempts (Fig. 1b). Of the other most common forms of self-injury in autism, behaviors of hitting the self (engaged in by 32 participants), punching objects (engaged in by 33 participants), or severely scratching or pinching (engaged in by 56 participants) were not significantly predicted by suicide ideation or attempts.

Among individuals who self-harmed, there was no significant relationship between the distress participants felt at self-harming and their responses to the suicidality question (*p* = .152). There were, however, significant correlations where higher scores to the suicidality question (where 1 reflects “never,” 2 reflects “suicide as a passing thought,” 3 reflects suicide ideation and 4 reflects an attempt) were associated with greater use of NSSI for regulating low-energy states (*r*_s_ = .371, *p* = .001), or for punishing oneself or deterrence from suicide (*r*_s_ = .379, *p* = .001), or for the purpose of sensory stimulation (*r*_s_ = .319, *p* = .005).

### Relationships between suicidality and other risk variables in self-harmers

Of the other variables which were hypothesized to correlate with higher scores for the suicidality question, only the range of self-injurious behaviors was associated with higher scores on the suicidality question (*r*_s_ = .419, *p* < .001). Participants who had engaged in NSSI for a longer time tended to have higher scores on the suicidality question (*p* = .075), and those who had a greater lifetime incidence of NSSI were more likely to score highly on the suicidality question but not significantly so (*p* = .151). There was a negative trend where participants with greater perceived social support tended to score lower on the suicidality question, but this was marginally non-significant (*p* = .078).

## Discussion

Self-injury, with its close associations to suicidality in the general population [6–15], is much neglected in autism research, as is its relationship to suicidality. Our data corroborates and expands on a key finding from one existent report in autistic people [53]: that suicide ideation and attempts were significantly associated with self-injury, and more prevalent in those who self-injure.

Further exploration of this relationship was motivated by questions raised in our previous study of NSSI in autism [40]. We were intrigued by the dichotomy between individuals greatly troubled about their self-injury and those who seemed to accept it matter-of-factly. With the ultimate goal of identifying red flags that might distinguish self-harmers at greatest risk of suicide ideation and attempts, we queried whether, as implied in our previous qualitative and quantitative data, some cases of self-injury might be relatively benign. Though the present data is preliminary, it negates any complacency that onlookers might feel about self-injury. Here, efforts to uncover the relationship between self-injury and suicidality found no relationship between how positively participants viewed their self-injury and their likelihood of suicide ideation or attempts. In other words: how participants felt about their self-injury appeared to be of little importance in decreasing the risk of suicidality posed by NSSI. This is consistent with the theoretical viewpoint that regardless of feelings about it or its functional purpose, NSSI increases pain tolerance and decreases fear of pain and death, thus creating the capability for lethal behaviors in those with pre-existent suicide ideation [16, 17, 20–23]. This could explain why the duration of NSSI, in our analysis, was not significantly associated with the risk of suicidality, though there was a trend in this direction. Just having partook in self-injury, current or historic, was predictive of suicide ideation or attempts.

Though the data suggested that an individual’s feelings about self-injury may not accurately reflect its seriousness, it also indicated that the relationship between NSSI and suicide ideation/attempts may be influenced by (a) the *features* of self-injurious behavior, and (b) the *functional purposes* which drive NSSI. With respect to the former, our analysis suggested that certain self-injurious behaviors may be of heightened concern to clinicians. In particular, suicide ideation and attempts were seen significantly more frequently in cutting than non-cutting participants. This observation has also been made in non-autistic self-harmers [14], and theorists posit that the more painful and physically damaging self-injurious behavior is, the more it decreases inhibitions and increases capability for suicidality [16, 17]. This is consistent with our findings around the three other most common forms of self-injury in our autistic sample (hitting oneself, hitting objects, and scratching/pinching), which unlike cutting were unrelated to the likelihood that participants had engaged in suicide ideation or attempts. In the same vein, a more diverse range of self-injurious behaviors, which in non-autistic people tends to imply more *extreme* behaviors [16, 17], was related to greater likelihood of suicide ideation and attempts in our sample.

As concerns the effect of the *purposes* of NSSI on suicide risk, significant correlations were seen between suicide ideation/attempts and use of NSSI for regulating low-energy states and for self-punishment or deterrence from suicide. These relationships mirror the close relationships seen in the general population between self-injury, suicide, and depression [6–8, 54–56], which is indeed a low-energy state especially characterized by self-punishment and self-criticism. The use of NSSI for communication and expression, and for regulation of high-energy states like agitation, anger, and anxiety, was not related to suicidality. We did, however, also observe a relationship between suicidality and NSSI for the purpose of sensory stimulation. Theoretically, one might link this to habituation and tolerance of pain which is one pathway through which self-injury might increase the risk of suicidality [14].

If the functional purposes that drive self-injury do serve as some indication of particular suicide risk, it might be also relevant to look at the particular *intrapersonal factors* which predict use of NSSI for different functional purposes—the goal of our previous study [40]. There, alexithymia emerged as a variable of particular interest in self-injury [40], perhaps unsurprisingly given its links to self-injury and suicidality in the general population [70, 71]. Alexithymia was particularly associated with the use of NSSI by autistic people to regulate high-energy states such as agitation, anxiety, and anger—but notably, the present analysis showed that engagement in NSSI for this purpose was *not* associated with increased suicidality. This could suggest that autistic people with comorbid alexithymia might be more likely to self-harm to regulate high-intensity states, but this use of self-injury does not incur especial risk of suicidality, besides any acquired capability generally accrued through self-injury [20, 21, 23]. It might be further extrapolated that alexithymia may be a useful predictor of self-harm but not suicide in autistic people, perhaps due its general ubiquity within this population [72]), but as the present study did not collect data on alexithymia alongside suicidality, this interpretation is highly speculative at present and requires further scrutiny. Another intrapersonal feature predictive of self-injury in our previous study was sensory sensitivity [40], which is consistent with the finding that autistic children use NSSI to regulate hypo- and/or hyper-arousal [50]. This seems somewhat discordant with the finding, in the present study, of a relationship between the use of NSSI for sensory *stimulation* and suicide ideation or attempts. A relationship between autistic sensory sensitivity and the use of NSSI for sensory stimulation would bridge this apparent contradiction, but this relationship was *not* significant when scrutinized in our previous paper [40]. Although this may relate to limitations of the methodology and power in the previous study, it may be that as with alexithymia, autistic sensory differences might be too ubiquitous to serve as significant predictors of suicidality. Additionally, it may be that individuals who use NSSI for sensory stimulation are driven more by the *correlates* or comorbidities of sensory differences rather than the sensory differences per se. Such correlates, in autism, include sociocommunicative abilities, insistence on sameness, cognitive problems and inattention, adaptive behavior, and as previously mentioned, anxiety and other forms of affective difficulties [41–45]. Notably, impulsivity is an intrapersonal factor associated with sensation-seeking through NSSI [73] and with acquired capability for suicide [74], and has indeed been linked to self-injurious behavior [75] and to suicidal acts [25] in autistic children. These speculations invite multiple further lines of enquiry, but we are also reminded of the complexity of self-injury and suicidality as interconnected but distinct phenomena. It seems likely that some individuals are more or less vulnerable to suicidality over and above being self-harmers, but the additional variables which predict suicide risk may not be entirely identical to those which predict engagement in self-injury.

Finally, we also examined one interpersonal feature that might affect the suicide risk of self-harmers: social support as measured by the ISEL-12. The data revealed that, worryingly, those self-harmers with greater social support were not significantly less likely to engage in suicide ideation or attempts, despite a trend in this direction. This may link, again, to the time-invariant risk posed by NSSI in decreasing inhibitions to suicide. It may be that the social support at hand is inadequate to ameliorate the challenges faced by this population, but one might also query the adequacy of our measurement tool—whether specific types of social support not captured by the ISEL-12, such as the sense of community with other autistic people [76, 77], might have been moderators of suicide ideation and attempts in our participants. This is indeed indicated by other reports where loneliness and the lack of social and practical support, possibly via the mediator of depression [78], are important features in the suicide risk of autistic people [53, 79]. Autistic adults themselves have testified to the life-changing and life-*saving* power of appropriate support in helping them move away from self-injury and suicide [80]. The alarming lack of confidence that agency workers, clinicians, and community mental health staff feels around providing this support [81] further highlights the crucial need for development in this area.

### Limitations and directions for future study

The current report raises important questions around NSSI and its relationship with suicidality, namely whether how people *feel* about self-injury reflects the risk that their self-injury might pose for future suicidality, and whether certain forms of self-injury should warrant more urgent attention. We provide a first attempt to address these questions, but there is a critical need for future research in this area, given the limitations of the current report. These include statistical limitations in the operationalization of variables, which often necessitated non-parametric tests. Non-parametric tests have a valid use in research; they allow for comparison of ordinal and nominal data, do not assume normal distributions, and are less swayed by outliers. However, they neglect certain characteristics of the data, such as averages and standard deviations, they lack power when a sample size is especially small, and in such cases, they are less likely to reject a false null hypothesis (type II error). Our sample was of a reasonable size, our findings in the main showed very low *p* values and tended in the direction one would expect based on previous investigations. However, especially when it comes to our operationalization of how individuals *felt* about their self-injury, our analysis may have lacked the power and detail to pick out those among the self-harming group who might be more or less vulnerable to suicidality. This extremely complex goal was motivated by observations from a rich qualitative analysis of individual responses, whereas the present approach utilized only quantitative data in a non-parametric approach.

In the present study, we did not examine distinctly whether self-harming individuals were more prone to suicide *ideation*, suicide *attempts*, or both. It may be important for future methods and analyses to distinguish more clearly between suicide ideation and attempts. The interpersonal model of suicide suggests this is a crucial distinction [20], particularly in identifying the factors that facilitate an individual’s transition from suicide ideation to suicide readiness and attempts—and this, in autism, is absolutely imperative.

One limitation of our report is its focus exclusively on autistic individuals without intellectual disability. These are in fact the individuals most at risk of suicidality [26, 30], but nevertheless it means that our data on the relationship between self-injury and suicidality cannot be generalized to autistic individuals with intellectual impairment. The data does suggest that certain forms of self-injury, such as cutting, are particularly associated with suicidality, as opposed to hitting the self, punching objects, or scratching or pinching. If autistic people without intellectual disability are more likely to engage in these kinds of activities than people with intellectual disability, this may be a factor in explaining their increased risk of suicidality; however, relationships between self-injury and suicidality in intellectually disabled autistic people or those with more severe learning disabilities are yet to be explored.

The generalizability of the present study is further limited by biases that may have been introduced by our sampling method: sending out email invitations, advertising our study in person at local support groups (this accrued the smallest return), and advertising it on social media. A number of studies have documented that women are generally more likely than men to engage with online surveys [82–84], and our disproportionate ratio of females to males would seem consistent with this observation, despite its incongruence with the elevated ratio of males to females diagnosed with autism [85, 86]. Notably, studies concerning mental health in non-autistic people have linked the lower up-take by men to the greater stigma that men experience around discussing and seeking help for mental ill-health [87–92]. It may be that autistic men may experience the same difficulties discussing mental health as their non-autistic same-sex peer group, which could have deleterious effects on their wellbeing.

Though our limited male sample prevented us examining the self-injury/suicidality relationship in men and women separately, sex is likely to be another variable of interest, given the modification of autistic presentation by sex [93], and the differences seen in presentation and diagnosis of mental health issues in autistic men and women. Our previous research suggests that autistic men and women are equally at risk of self-injury, though others suggested greater prevalence of self-injury in autistic women [35]. At present, it is still uncertain whether autistic men or women are at greater risk of suicide, and whether sex differences in suicide rates could further differ depending on the presence or absence of intellectual disability. Attempts to address this may be somewhat confounded by the lower recognition rates of autistic women without intellectual disability [94]. In the general population, death by suicide is generally higher in neurotypical men than women [95]. Closer examination, however, shows that neurotypical women are equally or *even more* prone than men to suicide ideation and attempts [95], which appears to be the case in ASC, too [53]. In autism, there may also be sex differences in the likelihood that male or female self-harmers might proceed to act on suicide ideation. Possible sex differences in the ideation-to-action transition might pertain in part to differing uses and features of NSSI in autistic men and women, which have not been addressed by previous studies of NSSI in autistic adults without intellectual disability [35, 40]. In non-autistic samples, there are some differences in the features of NSSI (for instance, wall-punching being a more common and often under-recognized form of self-injury in men [96], and cutting being more common in women [97]), and in the functional purpose of self-injury (with men less likely to use NSSI to influence others or avoid interacting with people [98], though others contradict this [4]). Sex differences in autistic NSSI, suicide, and the ideation-action transition are certainly a worthy line of enquiry. Future research should also differentiate between sex and gender, given the prevalence of gender dysphoria in autism [99, 100] and the high incidence of NSSI in non-autistic people with gender dysphoria [101, 102], who commonly experience high degrees of mental distress [103].

In attempting to identify autistic people at greatest risk of suicidality, our report focuses on self-injury as a particular risk factor, but a full and comprehensive assessment of the suicide risk faced by any self-harming autistic individual would need to recognize a number of other intrapersonal and extrapersonal risk factors related to suicide ideation and attempts in autism [24–28, 31, 32, 53, 104]. These include behavioral problems (in children); bullying and victimization at different life stages; increased likelihood of physical and sexual abuse; unemployment and financial insecurity; and, as previously mentioned, social isolation and failure in relationships. Features of autism including poor interpersonal skills, impulsivity, concrete and restricted thinking, reduced ability to make and change plans, and to cope with environmental changes, have also been associated with suicide risk—as has the strain of camouflaging these difficulties. As pertains to experiences like being bullied, victimized and abused, it is possible that self-injury mediates the effect of these experiences on future suicide risk, given that they also increase the risk of self-injury [105]. These experiences may also be relevant to the high rates of psychiatric illness in autism, such that it is not always possible to attribute the heightened suicide risk to autism alone [26]—depression and post-traumatic stress in autism have themselves been identified as serious risk factors [24, 28].

It is important to note that the present study did not control for the psychiatric comorbidities that were seen to differ between self-harming and non-self-harming groups. The differences (and indeed *lack* of differences) that we observed between our two groups may reflect sample disparities in our unequal numbers of self-harming and non-self-harming participants, and so should be taken with a note of caution until replicated in larger samples. However, participants with anxiety and other comorbidities were significantly more likely to be found in the self-harming group, though the groups were matched for depressive symptomatology. This may be indicative that psychiatric conditions in autism may be especially important for understanding and differentiating between NSSI and NSSI accompanied by suicide ideation and/or attempts. There are certain conditions that are elevated in the autistic community, such as eating disorders [106, 107], which might be especially significant in the context of assessing risk for self-injury and suicidality [108, 109]. For researchers, distilling particular *elements* shared between autism and other conditions associated with NSSI would be valuable. Poor interoceptive awareness, for instance, is shared by people with eating disorders [110], autistic people [111], *and* self-harmers and people who report suicide ideation or attempts [112]. In this study, suicide-attempters were less likely to ignore or distract themselves from painful sensations and were less able to regulate distress by paying attention to bodily sensations [112]. Interoceptive “disconnect” with the experience of pain would, theoretically, make self-harmers capable of severely hurting the body much as an object; that they exhibit both higher tolerance for pain *and* decreased fear of pain is consistent with the central role that both play in acquired capability for suicide [113]. Interoception is closely related to (or indeed an aspect of) alexithymia [114, 115], a feature of both eating disorders and autism. Interoceptive awareness of one’s “visceral afferent information” [111] is also, of course, closely related to differences in the processing and integration of externally-generated or exteroceptive sensory experiences [116]. As such, the exteroceptive processing differences (and indeed alexithymia) observed in our self-harming sample [40] might have been accompanied by interoceptive differences which could contribute to self-injurious behaviors.

This brings our focus, full circle, back to highlighting and teasing apart the contribution of these variables [40], among others, that might link NSSI as a transdiagnostic phenomenon, and might have relevance to self-harmers who attempt suicide. While researchers undertake this task, some have suggested that autistic children and teenagers should be routinely screened for suicide ideation, suicide attempts, and underlying psychopathology [26, 31]. The high prevalence of suicidal ideation and attempts in those diagnosed in adults suggests that these individuals, too, might benefit from screening at the point of diagnosis [24]. Clinicians are in crucial need of appropriate tools for this purpose, especially given their anxiety about treating mental ill-health in autistic people [81]. The development of brief autism-specific screening tests for suicidality is fortunately underway [117], and we laud this important effort.

As a final note, the relationship between NSSI and suicidality suggests that one route to reducing suicide risk might be to target initial engagement in NSSI. One recent model [5] acknowledges the “benefits” (i.e., functional purposes) of NSSI which, the model suggests, would be accessible to any individual if they were to overcome the “barriers” that prevent most individuals from ever trying NSSI [5]: lack of awareness of or exposure to NSSI, having a positive view of oneself (and thus reduced likelihood of self-criticism), aversion to physical pain and to stimuli associated with NSSI (such as blood), and sensitivity to social norms and negative reactions to NSSI. According to this approach, common antecedents to NSSI (such as negative self-image, the desire or need to communicate something via NSSI and the desire to affiliate with a group) reduce the aversive power of these barriers. Once a person has first tried NSSI, approximately 50% of individuals continue to engage in it regularly or with high frequency, having evoked an “affective engine” which simultaneously amplifies its benefits and reduces the barriers for further self-injury (for instance, the relief from pain may reduce aversion to the sight of blood). With consideration of an individual’s first experiences of self-injury, this model invites query regarding the factors that distinguish those who become regular users of NSSI from the large proportion who do not. With relation to autism, one might question whether or to what extent autistic individuals experience the same benefits and barriers to NSSI. Differences in the perception of pain [118–122] may suggest that physical pain is not aversive in the same way; similarly, one might query whether autistic individuals also possess a natural aversion to stimuli associated with NSSI, as studies of physiological response to “emotional stimuli” have found varied results [123]. In understanding autistic self-injury, it may be beneficial to adopt a similar transitional approach such as has been formulated in “ideation to action” [22] models of suicidality—a holistic overview from the risks and barriers that increase and decrease the likelihood of NSSI ever occurring, to the motivation for a first incident, and, from there, the sparking and maintenance of the engine which could build suicide capability.

## Conclusion

Despite previous reports of individual differences in experiences and perceptions of self-injury in autism, this study confirmed a clear relationship whereby suicide ideation and attempts predict the presence of self-injury, regardless of how participants perceive their self-injury. There is some indication that particular methods, such as cutting, and particular functional purposes of NSSI may pose a specific concern, as does increased diversity of self-injurious behaviour. Future research is imperative, however, in validating these preliminary findings and identifying variables which, in autism, increase the risk of NSSI and of NSSI which escalates to suicide attempts.

## Data Availability

The datasets used and/or analyzed during the current study are available from the corresponding author on reasonable request.

## Abbreviations

NSSI: Non-suicidal self-injury
NSSI-AT: Non-Suicidal Self-Injury Assessment Tool
SBQ-R: Suicide Behaviors Questionnaire Revised

## Footnotes

1 By using functional behavioral language to describe the “reasons” and motivations behind self-injury, we attempt to capture the fact that an individual may not make conscious or reasoned decisions to self-injure, and, indeed, that they may not fully understand why self-injury appears to help them. As used by Nock (2010), this terminology coaches self-injury in behavioral terms where antecedents and consequent events, through which behaviors are reinforced and maintained, are highly relevant. This approach, the author argues, lends itself to developing interventions.
